# radR: an open-source platform for acquiring and analysing data on biological targets observed by surveillance radar

**DOI:** 10.1186/1472-6785-10-22

**Published:** 2010-10-26

**Authors:** Philip D Taylor, John M Brzustowski, Carolyn Matkovich, Michael L Peckford, Dave Wilson

**Affiliations:** 1 Department of Biology, Acadia University, Wolfville, Canada; 2 Canadian Wildlife Service, Environment Canada, Ottawa, Canada; 3 Electro Marine Communications Inc., Oakville, Canada

## Abstract

**Background:**

Radar has been used for decades to study movement of insects, birds and bats. In spite of this, there are few readily available software tools for the acquisition, storage and processing of such data. Program radR was developed to solve this problem.

**Results:**

Program radR is an open source software tool for the acquisition, storage and analysis of data from marine radars operating in surveillance mode. radR takes time series data with a two-dimensional spatial component as input from some source (typically a radar digitizing card) and extracts and retains information of biological relevance (i.e. moving targets). Low-level data processing is implemented in "C" code, but user-defined functions written in the "R" statistical programming language can be called at pre-defined steps in the calculations. Output data formats are designed to allow for future inclusion of additional data items without requiring change to C code. Two brands of radar digitizing card are currently supported as data sources. We also provide an overview of the basic considerations of setting up and running a biological radar study.

**Conclusions:**

Program radR provides a convenient, open source platform for the acquisition and analysis of radar data of biological targets.

## Background

Biological use of radar originated in the 1940s when early users attributed echoes of unknown origin ("angels") that they observed on radar screens, to birds [1]. In the years since, zoologists have used radar to study the behaviour of many types of mobile organisms including birds, bats and insects [2]. Today, researchers study the movements of organisms using a wide variety of types of radar from small marine units [3] surplus military equipment [4] fixed beam "entomological radars" [5] to broad-scale Weather Surveillance Radar (WSR) arrays [6]. Radar is an especially useful tool where direct observations of biological phenomena are challenging, such as at night or in fog.

In spite of the relatively long period of time that radar has been available for use in biological studies, there are still major barriers to its more general use. One of these is the lack of an automated, cost-efficient tool that enables users to obtain digital radar data of known quality for a given project. For example, some use non-automated means for data collection such as video-taping and marking acetate sheets with putative tracks; such approaches are time consuming and prone to errors of omission and interpretation. Furthermore, non-automated approaches mean that it is not straight-forward to estimate error and bias (and correct for it) and so do not produce results that are comparable across studies. A range of 'in-house' solutions to automated data collection exist but are not readily available for use perhaps for cost or proprietary reasons. In general, there is a lack of ability within the research community to compare and validate various systems and studies, which in our view has hampered the broader development of radar zoology.

## Implementation

Here we present an open-source software program - "radR" - that addresses this problem. Program radR is capable of reading, extracting biological information and saving data from a digitized radar signal. Archival data formats allow for future inclusion of arbitrary additional information while preserving forward and backward compatibility. We present an overview of the current state of program radR and some additional material that outlines technical details about how to set up a small marine radar in what would be typical use for the radR software (see Additional file 1). For an in-depth overview of many of the details of radar terminology and function, its range of applications, and the strengths and pitfalls of the technology for zoological work the reader should consult Larkin [7]; for details of radar signal processing and tracking, readers should consult any of the more accessible radar texts (e.g. [8]).

Our ultimate aim is to increase the accessibility of radar technology to researchers, the consulting community and citizen scientists, in order to improve our ability to calibrate and compare results across studies [9-11] and to stimulate hardware and software developments in the field.

## Results and Discussion

### General layout and orientation of radR

Program radR provides a convenient platform for visualizing and recording radar data, developing and testing new algorithms, and evaluating and comparing radar hardware. It is intended as a research tool, and is not designed to be used for critical functions such as navigation, air traffic control or bird strike avoidance. It provides a baseline standard that offers an opportunity to increase the reproducibility and comparison across studies of ecological phenomena using radar. Program radR was developed for marine radars operating in surveillance mode (that is, where a radar beam sweeps repeatedly through some volume of space - a 'scan'), and has been primarily tested using two types of small marine radars (Furuno 1954/64 BB). However, it has been designed to be flexible and extensible, and so we anticipate it will be useful for a wide variety of other radar types, including different brands, antenna types and orientations, and wavelengths.

The program is written in C and R [12], an open source, statistical programming language that provides rich extension capabilities and a broad spectrum of statistical tools for post-processing data. Program radR runs under both Windows (XP/Vista) and Linux systems and is not vendor-specific except when interacting with proprietary radar digitizing cards of which two brands are currently supported. The core of the radR program is a 'processing manager' that consists of a user interface loosely based around the familiar media-player paradigm, that allows the user to start and stop processing, choose sources and sinks for data and output, and display data and the output from basic target finding (clutter learning and target extraction) and tracking ("track-while-scan") algorithms.

**Figure 1:**
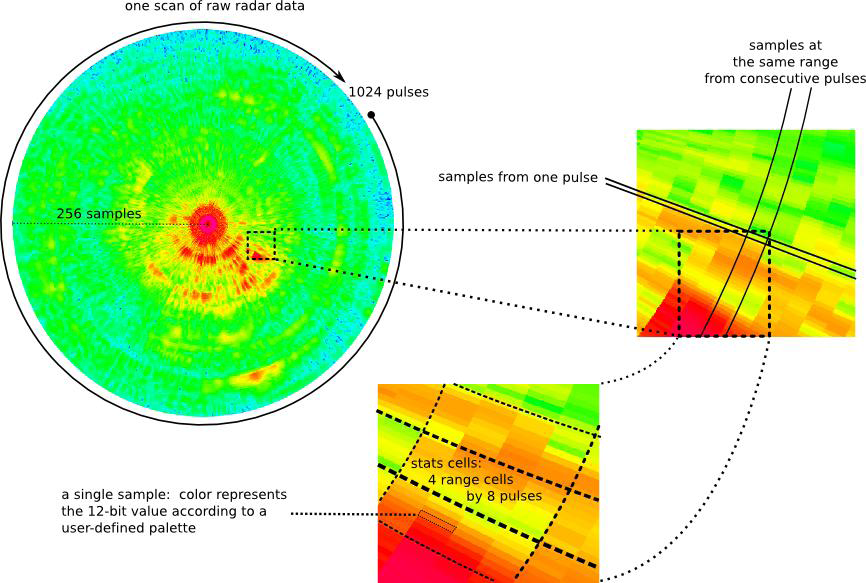
**Terminology used in radR for referencing the elements of a digitized radar scan **. The example shows a single 'scan', divided into 1024 pulses, each divided into 2048 samples. Each sample has a value from 0 through 4095, displayed on a coloured scale (blue low, red high). A 'stats' cell is the unit used to calculate means and variances of sample values for blip extraction (see Figure 2 and text for details).

The basic data object in radR is the "scan", which is a matrix of integers representing the power received by the radar at a set of uniformly-spaced sample times within each pulse of a sequence evenly-spaced through the radar's rotation (see Figure 1). Additional data, called "meta-data", denote conditions under which the scan was obtained, such as the time of the first pulse, the physical location of the radar (when available) and the radar's pulse length. Much of the work of radR consists of processing the scan matrix, to remove noise and clutter and extract putative targets. This processing is done in stages, and at check-points between stages (called "hooks") user functions written in R can be called to use or modify the intermediate data. Functionality in the program is delivered by a set of modules ("plugins") that implement functions called at some of the hooks. For example, a 'hook' is present at the point where all of the meta-data for a scan has been acquired from the data source. A plugin may then add additional meta-data for which it is responsible, e.g. the angle of the beam above the horizon. For a particular radar set-up, this information might be constant, or it might vary from scan to scan. Subsequent processing stages will use the data and metadata which might have been modified by a plugin at an earlier hook. The plugin architecture allows users to write extensions to the program in "R", without having to resort to low-level programming in "C". We believe that allowing users to customize the program using the same programming language that many of them will subsequently use for statistical analysis of the data it gathers, significantly lowers the barrier to use of radar technology. A current list of plugins can be found at http://www.radr-project.org. Much of the R code is verbosely documented in the source distribution (comments are stripped from files in the binary distribution).

At present, plugins can be grouped as follows: 1) acquiring data from a radar or archive file,2) adding or manipulating meta-data 3) processing, describing and saving the input data, 4) creating tracks and exporting data, 5) displaying information about the targets, and 6) other utilities. Here we specify the approach used within radR to accomplish each of the tasks above; details are evolving and are documented at http://www.radr-project.org. In the future we plan to migrate the project to either sourceforge.net or r-forge.r-project.org, and to take greater advantage of the R project's well-developed package management and distribution tools.

### Acquiring the radar signal

There are many considerations in choosing a radar and antenna and setting it up. These are generic to all studies, but are critical to obtaining useful data. We outline the major steps involved and some basic radar terminology in Additional file 1. Once the user has a suitable radar system, they require a means to obtain basic information from the radar, including converting the analog signal containing information on targets to a digital signal for processing. The problem is non-trivial, largely due to the huge bandwidths involved [[8]; Chapter 1], and so specialized radar digitizing cards are used.

radR has primarily been developed using the Rutter Technologies (St John's, Newfoundland, Canada) Sigma S6 radar digitizing card. A plugin allows the user to interface with this card, and to control, via Rutter's proprietary software, the sampling rate and maximum range of the card. At present, pulse length and Pulse Repetition Frequency (PRF) are set by the radar and inferred using tables provided by the user. (Readers not familiar with these terms should consult Additional file 1, some of the key radar overview papers cited therein, and a text on basic radar principles e.g [8]). A similar plugin has been written to acquire data from the Russell Technologies (Vancouver, Canada) "XIR3000C card). It is worth noting that plugins can be written for other digitizing cards, but that they will almost certainly require some "C" code, as vendor interface libraries will typically use data-types and calling conventions beyond those available in "R". A template plugin is provided which documents the requirements for a new digitizing card plugin, as well as providing the high-level R code skeleton (which would presumably call new "C" code, specific to the digitizing card).

For the advanced user, there is detailed documentation on how to convert an archive of unprocessed radar data into one of radR's own data formats, so that a user can use radR to post-process data collected by a different system.

### Processing the radar signal and extracting putative biological targets

Once a scan of radar data has been acquired, radR's top-level processing manager calls internal "C" functions to process it. Processing works as follows. The radar digitizing card or other source has provided a scan of data. As discussed above, this consists of a matrix of integers. Each column in the matrix represents a time-series of power received by the radar antenna in a short time-window after the radar has transmitted a single pulse of microwave energy. Individual numbers in the column represent the amount of microwave energy reflected back from within volumes of space at increasing distance from the radar, as well as noise from various sources both within and outside the radar system. The columns form a sequence spaced uniformly around the radar's plane of rotation (e.g. sweeping from 0 through 360 degrees of azimuth). Each row in the scan matrix corresponds to the energy received from a given "range cell", with individual numbers representing the energy received while the radar was pointing at a particular azimuth (or more generally, direction). For each *pulse *of energy transmitted by the radar (or at least for a uniformly spaced subset of pulses from one revolution of the radar), the return echo is digitized into a specified number of "samples" at a specified rate. Each sample thus represents the intensity of the return echo from a single pulse, for a given range cell. The possible number of samples and the resolution of the return echo intensity obtained depends on the digitizing card. For the 12 bit card which has been used for most work with radR, a sample is an integer in the range 0 to 4095, (i.e. 2^12^-1), the available digitization rates are between 5 and 60 million samples per second, and roughly 4 million samples per scan can be obtained. The user has some choice over how many pulses are digitized and how many samples are obtained per pulse, subject to the product not exceeding 4 million. Roughly speaking, the digitizing rate affects the ability to discriminate targets in the same direction from the radar but at different distances, as well as the precision of estimates of target distance; the number of pulses affects how many echoes will be received from a target in each scan, as well as the precision of estimates of target azimuth; and the samples per pulse, when divided by digitizing rate, controls the maximum range from which target echoes can potentially be received. Each scan can be subsequently displayed, stored or processed, depending on the user configuration.

The user can save all of the samples from each scan (in a 'raw' archive) but such files are massive (e.g. about 5 GB for every hour of recording, depending on the digitizing parameters). This can be reduced significantly in situations where only a fixed portion of each scan is of interest to the user (e.g. when the radar is monitoring off-shore activity from the edge of a body of water, where only data from, say, 0 to 180 degrees azimuth is desired), or when the user is able to set a noise threshold such that sample values below it are discarded (either by adjusting the digitizing card controls, or by a radR parameter). In these cases, a standard lossless compression algorithm can be (optionally) applied to reduce the size of recorded files,

Normally however, radR is configured to extract and save a subset of information from each scan - the parts that contain putative biological targets ('blips'). These smaller archives ("blipmovies") provide an archival record of the observations that can be subsequently viewed and analysed (see below). Blipmovies typically reach a maximum size of ~2 MB for each hour of recording (depending on the specified sampling rate and depth, and the numbers of biological targets that are detected).

**Figure 2:**
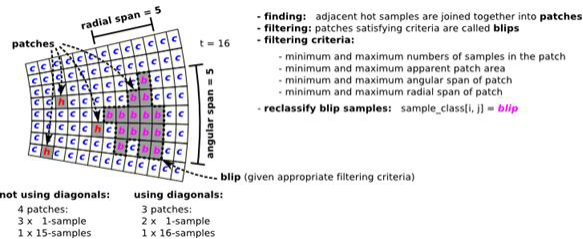
**Example and terminology used for blip extraction **. See text for details.

At present, radR extracts putative biological targets from the digitized signal in a simplistic way. For a user-specified number of scans the program computes a temporal mean and mean deviation of the strength of the radar echo from user-defined windows of samples and pulses across the entire scan. This is the background. The program then computes, for each subsequent sample, an intensity z-score for that sample (i.e. the intensity of the signal return for that azimuth and range cell combination, relative to the background distribution for its window). Samples that exceed some user-defined threshold in the z-score are considered 'hot' and are grouped with adjacent 'hot' samples into 'patches. Patches that satisfy user-defined filtering criteria (fixed values or arbitrary functions based on numbers of samples, PPI area, angular and radial span) are considered blips, and retained. These can be of any size above the user-defined minima described above. The learned pattern of background can optionally be updated with data from each scan, using a scheme that amounts to exponentially weighting data from previous scans at a user-specified decay rate. A diagrammatic representation of the process is presented in Figure 2.

The target finding algorithm was developed to loosely mimic how human radar operators appeared to detect targets, and raw data is saved for each blip so that physically realistic target detection models can be applied to them. The method does not work well in situations where there are rapidly changing patterns of 'background' such as reflections from surface water (waves) and during periods of rain. In these situations, the algorithm detects many 'non-biological' blips, some of which can be filtered on-the-fly using user-defined criteria. More sophisticated 'blip-finding' algorithms exist (e.g. [8,13]) that can make much better use of the information contained within the returned signal; we plan to develop these in future versions of the software.

At present, blips are stored and manipulated in the digitized units obtained from the associated radar scan converter (e.g. for the 12 bit Rutter card, a sample can theoretically have a value ranging from 0-4095). These sample values can be converted to estimates of the returned power for that sample by determining (either through the manufacturer's specifications, or through an empirical test of the conversion card using a microwave generator) and substituting the appropriate values or estimates into the radar range equation [8,10]. This functionality is card-specific, and is being developed as a separate plugin.

Users would normally save blips to a permanent archive (a "blipmovie") which is a file-backed structure that behaves like an R language "list" object. Blipmovies can be re-run, further modified (by filtering out blips) and processed. Typical processing steps include outputting files of blips (timestamps, x, y, z coordinates, and their associated characteristics) or combining blips into tracks. All processed files can be saved to text files, for processing outside of radR, or re-recorded as (presumably filtered) blip movies.

Another plugin provides important information to the program and to the archived movie regarding the basic characteristics of the antenna and scanner including the type of antenna ("t-bar" or "dish"), the beam height and width, the angle of the beam center off the horizontal, the angle of the scanner off the horizontal, and the geographic co-ordinates of the scanner.

Through another plugin, the user can apply different processing and filtering rules for different portions of the scanned volume, and can entirely discard data from specified sectors.

### Track compilation and exporting

A single target flying through the radar beam can be detected on multiple scans of the rotating beam, creating a series of blips. These blips can be linked together to form tracks (track-while-scan), so that the velocity and direction of targets can be estimated. Currently, radR provides two track-building models: one based on a simplistic nearest neighbour (NN) algorithm and the other based on a multiframe correspondence (MFC) algorithm [14]. Tracks can be fit in real time or to archived data.

The NN model is primarily used as the basis for the more sophisticated MFC model. It builds tracks by minimizing the distances between existing tracks and possible new blips ("extension distance"), subject to some constraints. It first computes the distance from every new blip in a scan to the last blip in every active track. For all such pairs, a speed, turning angle, and relative change in blip area or intensity (all user-defined) are computed, and pairs for which these values do not fall within user-specified ranges are discarded. Among remaining "feasible" pairs, blips are assigned to tracks in such a way as to minimize the total "extension distance"; i.e. generally by matching tracks to their nearest feasible blips, and settling conflicts according to the minimum extension criterion. For example, if a new blip is the closest feasible blip to two different tracks, it is matched to the closer of the two tracks, with a "coin toss" for ties. Blips which are left unmatched in one scan may be extended into tracks in a subsequent scan, provided this occurs before their user-defined "expiry" time. The model uses an algorithm from the Stanford GraphBase package of Knuth [15,16].

The MFC method is more robust than the NN method. It employs a non-iterative greedy algorithm for multiframe point correspondence as outlined by Shafique and Shah [14]. It begins with two scans, matching blips between them using the NN algorithm and then assigning a velocity to the set of matched blips. When third and subsequent scans are processed, the algorithm considers all possible matches between blips in the first two scans, and those in the third. A "gain" function returns the "quality" of match between each new blip and an existing track (or initial segment thereof). The default gain function is a weighted sum (expressed on a log scale) of two components: the proximity of the new blip to the next location predicted for the track by assuming constant target velocity; and the homogeneity of target velocity (not just speed) when the new blip is added to the track. Tracks are extended with (or broken and re-matched to) new blips in such a way as to maximize the total "gain". When subsequent scans are processed, the procedure is repeated. All possible track segments connecting the blips in the previous k-1 scans and the blips in the new scan are considered, optimizing between proximity to predicted position and consistency in velocity (k is a parameter chosen by the user). Tracks may be broken and attached to a new blip, in which case the blips from the broken-off track tails participate in a second track-building phase with any other unmatched points. In effect, tracks can be retroactively corrected back k scans to better match newly acquired blips. The procedure is flexible in that the gain function can be modified or specified by the user to change the nature of the track building.

In informal tests, the algorithm works well (e.g. detects most 'valid' tracks and does not create incorrect tracks) when there is limited 'noise' (e.g. blips that are actually rain or surface water), where tracks are long, and more-or-less linear, and when the density of targets is lower. We have not directly quantified errors in track-building at this time, since we are actively undertaking calibration experiments that will allow us to incorporate additional information from blips into the blip finding, blip filtering and track building algorithms, all of which we expect to improve track-building performance.

Other, possibly superior, methods for track building exist (e.g. Multiple Hypothesis Testing; see [17]) that could be implemented in the future by us or the interested user.

Summary data for each blip and(or) tracks can be exported to text files or raw R-format data files for processing outside radR. At present, exported data includes basic blip parameters (e.g. time, x, y, z) and summaries of blip characteristics (number of samples, area, "perimeter" in the PPI display, mean and maximum intensity, angular span, radial span) that may be useful for blip and track classification. When exporting data on tracks, each blip is associated with a unique track number. Complete information on blips (e.g. actual values for all samples within a blip) are retained in the blipmovies, and can be extracted by the user via additional plugins. (example blipmovies can be found at http://www.radr-project.org).

A simple 'batch' mode allows the processing of multiple files in a single job, controlled by a script rather than the by the graphical user interface.

### Displaying the targets

Biological targets (blips) are displayed in a "plan-position indicator" (PPI) plot window which the user can pan, zoom and rotate. The PPI displays a scan by plotting a spot at the nominal range and azimuth of each sample, with the spot's color depending on the sample value. A pointer can optionally display the current characteristics of any blip or track on display. The user can also provide a background image such as an aerial photograph of the area being sampled that can be translated, rotated, and scaled within radR to align with radar data.

**Figure 3:**
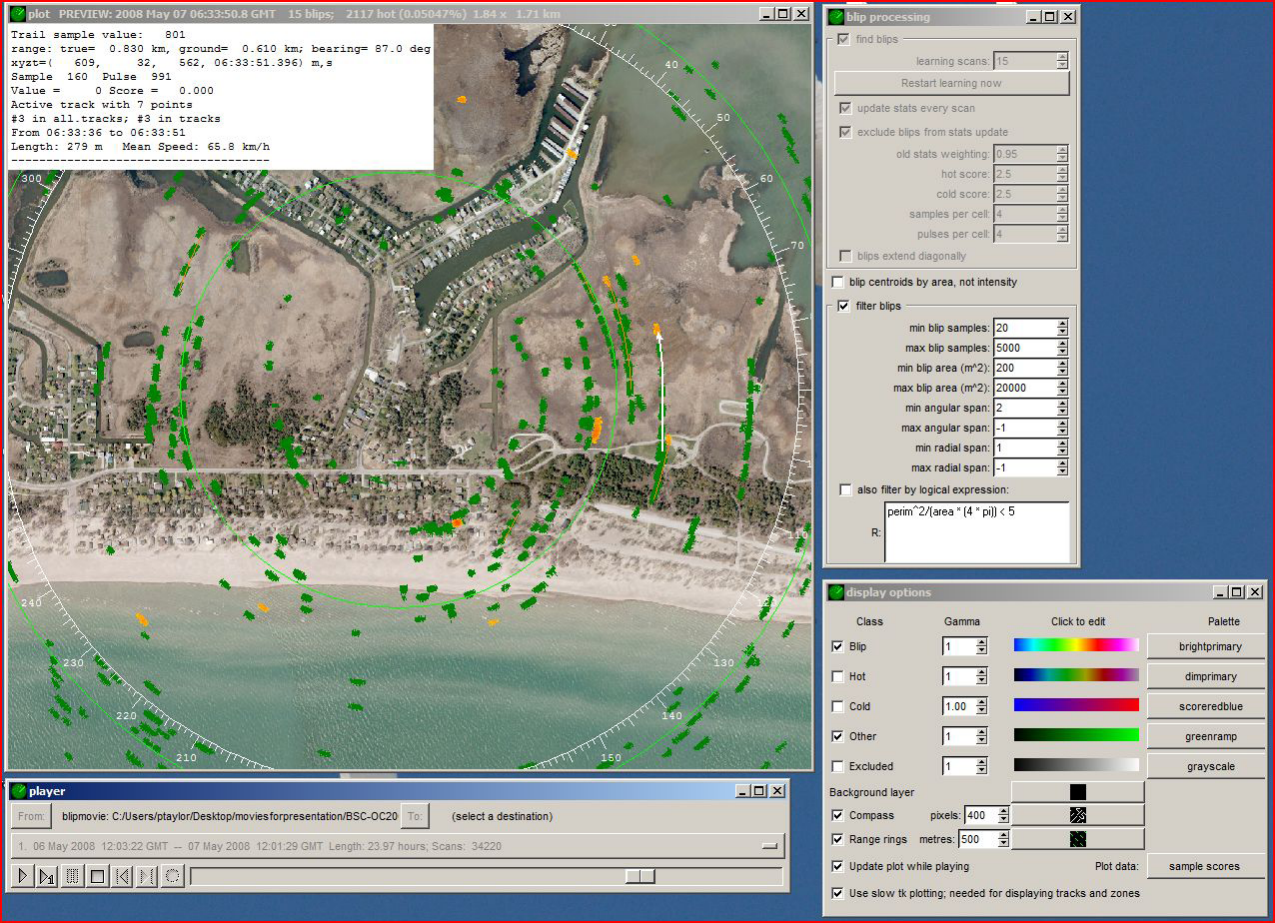
**Example program radR screen layout showing bird targets migrating overhead in spring at Old Cut Field Station, Long Point, Lake Erie, Canada**. The main 'plot' screen is in the upper left; the 'blip processing functions' in the upper right, display options in the lower right, and 'player' controls in the lower left. On the plot screen, an aerial photograph underlay is shown, with North at the top. Blips detected in the current scan are an orange colour; blips that are green show positions where targets were situated in previous scans. A single track (white arrow pointing N on the right hand side of the plot screen) is currently active and shows the relationship between a current blip (orange) and the previous locations of that target. Details of that track are shown in the text box in the upper left hand corner of the plot screen.

The user can mimic the appearance of a phosphor screen by allowing blips to persist and fade over multiple scans ('trails'). Figure 3 provides a screenshot of an example session with an underlay, several targets, and trails from those targets displayed.

### Other utilities

Plugins exist for a number of other purposes, allowing users to create demonstration 'clips' of movies in animated GIF format, to add comments to blipmovie archives at specific points, and to generate random blips with known patterns and characteristics that could serve various purposes, such as algorithm testing.

### Extensibility

Program radR is extensible by the user by writing "hook" functions in R, as discussed earlier. For example, each time the *tracker *plugin declares a track "complete" (because no blip has been added to the track for some user-determined number of scans), a user function, written in R, could be called with the coordinates of the blips on the completed track. This could then be used to apply a user-defined hidden-variables model for improving the estimates of target locations along the track, or to add the track to a 3-dimensional scene.

### Software testing

Software has primarily been tested in an ad-hoc way, under field situations (e.g. as part of ongoing research projects) and similarly, through post-processing of data. As such, some parts of the code are known to be more robust than others. Program radR is in active development, and new features are added regularly. We maintain a 'stable' release and development versions of radR available for download at http://www.radr-project.org.

### Using radR

Details of how to use radR in a field setting are provided at http://www.radr-project.org. These will change from time to time as new features are introduced, and bugs are corrected. The basic steps include: 1) Installing radR and the supporting version of R on the local computer; 2) configuring radR so that it is aware of the current setup (type and orientation of the scanner and antenna; 3) selection of parameters for the detection of blips (and filtering) and 4) Recording. In our use, we are liberal with the amount of data collected (that is, we acquire information on many putative targets that are almost certainly not targets, then filter them out during post-processing). Although it is possible (given sufficient memory and processing power) we do not usually attempt to process tracks in real time, leaving those steps for post-processing.

Once data are acquired, radR is used to further filter and create tracks. Filtering and track-building parameters are situation and radar dependent; individual users will need to calibrate their systems and view their archives to determine settings that meet their needs. Special consideration needs to be made for the classification of targets; radars with short wavelengths (e.g. the 3.2 cm wavelength of typical X band marine radars) readily detect birds, bats and insects and these types of targets are not easily separable (if at all) without additional information [10]. radR can facilitate target classification by providing the user with metrics such as target speed, height and direction. If a proper calibration protocol has been carried out (see Additional file 1) then radR can be configured to provide information on the radar cross sections of targets (RCS) or volume reflectivity.

## Conclusions

We have developed and extensively tested radR in field situations over the past five years. The software provides an open solution to the problem of acquiring and preparing for analysis radar data for use in biological studies. We hope that the provision of a widely applicable, freely-available open source tool will enable researchers working with radar data to improve and standardize the acquisition, storage and analysis of these data greatly enabling comparisons across multiple times and studies and further developments in the field. We further welcome other researchers to collaborate with us on the future development of the software.

## Availability and requirements

**Project name**: Project radR

**Project home page**: http://www.radr-project.org

**Operating system(s)**: Full functionality on Windows XP systems. On Linux systems, all functionality except the ability to read live data from radar digitizing cards because the proprietary cards supported so far lack Linux drivers

**Programming language**: R, C, C++

**Other requirements**: R 2.5.1, digitizing card interface library and possibly server program from one of the two currently supported hardware vendors.

**License**: GNU GPL Version 2 or later.

**Any restrictions to use by non-academics**: None

## Authors' contributions

PT developed the initial ideas, provided the overall conceptual framework for the project and is the main writer. JB wrote all of the code and formulated most of the internal conceptual organization for the program. CM and MP undertook extensive field testing, wrote sections of the manuscript, and provided key feedback on aspects of program and project implementation. DW provided extensive technical advice and modifications to hardware. All authors read and approved the final manuscript.

## Supplementary Material

Additional file 1 **A basic primer for implementing a radar study**. An outline of some basic information needed to implement a radar study Click here for file
