# Supplementary material for: radR: an open-source platform for acquiring and analysing data on biological targets observed by surveillance radar
Source: BMC Ecol. 2010 Oct 26;10:22. doi: 10.1186/1472-6785-10-22 (PMC2988708; doi:10.1186/1472-6785-10-22)
Supplement: Additional file 1 — A basic primer for implementing a radar study. An outline of some basic information needed to implement a radar study [file 1472-6785-10-22-S1.RTF]

Additional File 1
A basic primer for implementing a radar study
To get the most from program radR (or any other radar software), the technical elements of the radar setup must be sound. Comprehensive reviews of radar technology as applied to wildlife studies have been undertaken by Eastwood (1967), Williams et al. (1972), Cooper et al. (1991) and Bruderer (1997) and many of the practical applications of radar (particularly as applied to studies of bird migration) are well known (e.g Gauthreaux and Belser (2003)). Larkin (2005) provides an excellent overview of the use of radar techniques for biological studies and Ruth (2007) contains many useful practical perspectives. The user should become familiar with all of these documents. Our purpose here is to provide additional details for the specific problem of setting up a low-cost, small marine radar, for use in biological studies – what we would consider to be a typical situation in which a user would use the radR software.

There are three main technical considerations when conducting a radar study with a small marine radar: 
1)	Selecting a radar and antenna; 
2)	Setting up and tuning the radar;
3)	Acquiring the signal from the radar.

	1) Selecting a radar and antenna
Eastwood (1967), Bruderer (1997) and Larkin (2005) provide comprehensive overviews of the advantages of different types of radars for biological study.  The first question to consider is the type of data required by the user.  Of primary concern to most radar users are the maximum range at which a target needs to be detected and the minimum size (more correctly, the radar cross section; see Larkin 2005 for details) of a biological target that can be detected at a given range.  Estimation of these values requires use of the radar equation (Eastwood 1967, Larkin 2005, Richards 2005) and depend on at least three parameters: the gain of the antenna, the power output of the radar and wavelength of the radar signal.  In choosing a radar system, it is important to consider the non-linear relationships among key variables: e.g. doubling radar power will not double the maximum range at which a given target can be detected.  In fact, the radar equation implies that power received from a given target at a fixed distance grows only linearly with the power of the transmitter, whereas it grows with the square of the antenna gain.  Thus, choosing an antenna with a 40% higher gain has approximately the same effect on target detection range as doubling radar power.  Moreover, to a first approximation, either measure will only increase maximum detection range for the given target by 19%, due to the inverse fourth power dependency of returned power on range.

Antenna type and size
The measure of the ability of an antenna to focus its radiation in a narrow beam is termed gain (Eastwood 1967; Richards 2005). Gain is proportional to the square of size (width, length or diameter) of an antenna.  Higher gain (and so usually larger) antennas are required to detect targets at longer ranges (Larkin 2005). The simplest radar antennas produce beams that are characterized by a 'main lobe' (with the bulk of the radar's energy) and multiple 'side lobes' (e.g. see diagrams in Richards 2005). A primary feature of an antenna is the beam width and shape, where beam width is defined as the angular span between half-power points of the main lobe (Richards 2005). It is important to note that, depending on range and reflectivity, some targets will be detected beyond these half power points, and possibly within the side lobes of the radar, giving any particular antenna a wider empirical beam width. These effects may not be trivial (Shmaljohann 2008; and see more details below) 

The two main antenna types that have been used with marine radars for biological studies are the parabolic dish and the open array or "t-bar".  A parabolic dish produces a conical beam – depending on the focal length, for wavelengths of 2.5-3.75 cm (X band) a dish with an outside diameter of ~ 0.5 m usually produces a beam width of ~ 4°. The angular diameter of the beam decreases as the diameter of the dish increases (for a given focal length). When a beam produced by a parabolic dish is pointed at an angle above the horizon and spins through 360° ('scanning mode') the user can determine the geographic position of targets (x, y co-ordinates) and height (z) of targets using simple trigonometry. At low beam angles, relative error in x and y are low and relative error in z is high; at higher angles, the reverse is true. In contrast, an open array antenna generates a fan shaped beam that is narrow on the horizontal axis (~1-2°) and wide on the vertical axis (~20°). The horizontal narrowness of the beam is largely a function of antenna length, with longer antennas producing narrower beams. The open array provides positional information on targets but because the target may be anywhere within the wider span (~20°) it does not provide direct information on height (z) when used in its normal horizontal orientation. 

Open array antennas are the standard equipment for boats, and thus can be readily purchased from any radar manufacturer. There are few commercial manufacturers of parabolic dish antennas, and usually these must be custom made or bought from secondary markets (e.g. military surplus). It should be borne in mind that large antennas can be cumbersome to manage, more expensive, and more susceptible to wind disturbance.

Power output
The peak power output of a marine radar, measured in kilowatts, influences the maximum detection range of a target. More powerful radars often have larger motors, which means that larger antennas can be used. In practice, over a large range of radar powers, this means that detection range scales up faster than peak power output alone would suggest. However, zoologists tend to use smaller 'portable' radars with outputs between 3 kW (Williams et al. 1972; Peckford & Taylor 2008) to 50 kW (Gauthreaux and Belser 2003; Schmaljohann 2008) with the most commonly employed units ranging between 10 kW (Cooper et al. 1991, Harmata et al. 1999, Tulp et al. 1999, Petersen et al. 2006;) and 25 kW (Desholm 2003, Kahlert et al. 2004, Huppop et al. 2006), and within this range, the discussion about selecting a radar and antenna (above) applies:  doubling the peak output power only increases the detection range by roughly 19%.

Wavelength
Bruderer (1997) states that the detection of small birds is maximized with wavelengths 3.8 -15 cm which comprise the C band (3.8 - 7.5 cm) and the lower part of the S band (7.5-15 cm). However, the even smaller wavelengths of X band radars (2.5 – 3.75 cm) make the units better able to detect small targets such as insects (Bruderer 1997). Signals from longer wavelengths are not as influenced by precipitation (Richards 2005) and so may be better for observing biological targets under rainy or snowy conditions (Larkin 2005).  The trade-off is that the probability of detecting smaller targets of interest is likely lower with these higher wavelengths (e.g. S band); the effects of these trade-offs in field situations require considerable further study.

Target discrimination and antenna type
There are tradeoffs between open array and parabolic antennas due to the widths and different shapes of the beams. For both antennas, the accuracy of positional information increases as the beam width narrows, but motor size and wind-loading means that the maximum size of parabolic dishes is about 0.75 m and the maximum length of an open array antenna is about 2.5 m. Because they usually have smaller horizontal beam widths, open array antennas will usually provide better azimuth resolution and so can resolve targets that are closer together in azimuth. Because the beam is wide in the 'vertical' dimension, open array antennas cannot resolve targets that are at the same azimuth and range, but at different altitudes. Target discrimination is also a function of pulse length (see below), the resolution of the digitizing card and the angle of the antenna above the horizon and the user must consider all of these factors when interpreting the data acquired from any particular setup. Finally, open array antennas also scan a greater volume of sky due to the larger vertical beam width; parabolic dishes on the other hand, allow for easier positioning of the beam to scan particular heights or areas of interest, and provide better estimates of target height along with reasonable estimates of geographic position. 


Quoted beam widths from antenna manufacturers are approximate, and, for a variety of reasons, are not accurate representations of the volume of space sampled. There are at least three important considerations. First, quoted widths are the angular distance between the 'half-power points' – the points on either side of the beam where the return echo is half the peak output (Richards 2005). Thus, at a given distance, targets with larger radar cross-sections (i.e. that reflect more energy) will produce larger 'apparent' beam widths (Schmaljohann et al. 2008). This relationship is further complicated by the fact that radar cross sections do not vary as a simple linear function of the size of the object (see the diagrams and discussion in Larkin 2005 and elsewhere).  Second, the power of the return echoes decreases with the fourth power of target distance, so effective beam width decreases with increasing range, because target detection requires echoes stronger than the background. Third, since the power of the return echo is influenced by atmospheric conditions (Richards 2005) those conditions will also influence the 'beam width'. Collectively, these considerations mean that the expected relationship between the probability of detection of a target of a given size with distance is not simple, and needs to be calibrated for any given combination of site and radar. Furthermore, the means by which targets are identified and extracted from the background (e.g. the particular software settings or method used for target detection) will further influence these detection rates. Schmaljohann et al. (2008) outline some details of how to calibrate a radar for a given radar study and Zaugg et al. (2008) provide implementation details for a large tracking radar. Further details can be found in various radar handbooks (e.g. Richards 2005). These considerations also make target classification a challenge which requires considerable additional work (e.g. see comments by Gauthreaux 2007).

Calculating positions in space (x, y, z)
The height of a radar beam influences the accuracy of height estimates (z) (thicker beams have less accuracy).  For a dish antenna, this also varies with the angle of the antenna axis from the horizontal – height error decreases as the angle of the beam increases and is minimal when the beam is in the vertical position. For example, a conical beam with a quoted beam width of 4°, pointed at 45° off the horizontal, will produce estimates of z that are ~25 m higher or lower than the true z, when a target at 1000 m range is detected at the lower or upper “edges” of a beam, respectively. To acquire height information with minimal error, some users use open array antennas tilted at an angle, or on their sides (Harmata et al. 1999); thus, an object detected in a ~20° beam pointed straight up, will, at the half power points be about 10° off the vertical and at a range of 1000 m, introduce an approximate maximum error of ~15 m in z.  Note that in these examples, error in range is neglected.  In practice, range resolution is limited by digitization sampling rate (e.g. to +/- 5 m at 15 MHz), which is an additional source of error.  Information on height can also be recovered from an open array antenna provided there are multiple instances of a target (e.g. a track) and an assumption of constant height over the length of the track is made (Cohen and Williams 1980) but the uncertainty associated with these estimates is not well known. If an open-array antenna is tilted at a high enough angle (e.g. if it sweeps a cone) then the fact that the upper (inner) part of the beam sweeps a cone of smaller circumference than the lower part of the beam can be used to determine position in beam (with varying and poorly understood levels of error) which can be used to obtain an approximation of z.

Both height and spatial information are sometimes obtained by operating a vertically scanning radar simultaneously with a horizontal scanning radar (Krijgsveld et al. 2005) or alternating between the two positions with one antenna (Cooper et al. 1991), but the user cannot readily acquire simultaneous information on a single target in this way. Much more sophisticated methods of recovering information from targets and tracks, and combining information from multiple radars are possible; details can be found in various radar texts (e.g. Blackman & Popoli 1999). 

2) Radar setup and configuration
Like any specialized piece of equipment, a radar must be set up and tuned for optimal data collection.  The place to start is the manual for the particular radar you are using, but there are general considerations that should be taken into account when using a radar for biological surveys. We highlight what we feel are the most important here.

Radar Tuning
All new radars must first be tuned, optimized and calibrated.  A radar works by sending out a pulse, and measuring any returned signal (or “echo”) from objects in the beam throughout a set interval of time corresponding to a fixed maximum range.  Proper tuning is a fundamental part of maximizing the strength of echoes.

Marine X band radars emit electromagnetic pulses at a fixed (for a given model of radar) wavelength of approximately 3.2 cm. A pulse is emitted at or near the radar's nominal power for a short period (50 to 1200 nanoseconds) ,after which the transmitter switches off and the receiver listens for echoes at the same wavelength during some specified time window. The frequency at which this transmit/receive process is repeated (the “Pulse Repetition Frequency” or PRF) is 600 to 3000 Hz depending on the radar model and current maximum range setting.

In order to decipher the information contained in that return pulse, the pulse must first be amplified. To amplify a signal at such a high frequency and produce stable results at a reasonable cost is technically challenging. Marine radar usually employs a low cost solution based on the superheterodyne principal (Richards 2005). A stable oscillator source, similar in frequency to that transmitted, but displaced by a small amount, is located in the system receiver.  This Local Oscillator (LO) signal is connected to a device called a “mixer” where the returning signal is also introduced.  Sum and difference frequencies are produced in the mixer with the lower more easily amplified signal being filtered and chosen. This “Intermediate Frequency” (IF) signal is then amplified and the signal information is extracted at a detector device, which removes the IF carrier. The bandwidth of this receiver is usually made adjustable to provide for lower noise, improved performance depending on pulse width and range in use. Unfortunately due to temperature, mechanical and other constraints, the transmitted frequency will often drift.  In a simple radar set when such a drift is encountered, the mixing of the off-frequency return signal with that from the LO produces an IF value other than that to which the IF amplifier is optimally tuned.  The result is that detected signal performance quickly deteriorates. In modern receivers, an automatic tuning system is used whereby the LO tracks any changes in transmit frequency by monitoring an IF and thus increasing or decreasing the LO frequency so the result is always at the maximum IF value.

For example, Furuno products have an initial "Optimization" setup that establishes the characteristics of the IF tracking system. A standby "manual" tune function is also available in order to provide the operator with a second means of verifying correct operation of the receiver system.

Different radar manufacturers will employ different methods for tuning, optimization and calibration, but it is imperative that all are undertaken when first setting up a radar, and periodically throughout usage, to ensure optimal performance.

Radar settings: Gain, rain and sea clutter, IR rejection.
On most commercial radars users can adjust the image that is presented on the display screen (commonly termed a plan position indicator, or PPI) through various signal settings and filters.

Users can adjust the type of outgoing signal through changes to the pulse length and PRF. Pulse length is the amount of time between the leading and trailing edge of one transmitted radar pulse. Small marine radars generally have three pulse length settings (short, medium, and long) and pulse length is selected partly on the basis of maximum range from which the user wishes to obtain information. Longer pulses are necessary for detecting targets at more distant ranges but shorter pulse lengths provide better range resolution because their energy is 'spread out' over a shorter time (and distance) (Eastwood 1967, Larkin 2005). The range is limited by the choice of PRF. Selecting an 'ideal' range is really a matter of selecting a suitable PRF and PL and depends on the expected radar cross section of the targets a user is interested in at a particular distance. For example Cooper et al. (1991) suggest collecting data using short PL at a range of  ~ 1.4 km which, using a 10 kW X-Band radar allows for the detection of passerines. Considerably more empirical work is necessary to determine what pulse lengths are best for different types of studies.

User-applied transformations of return signals found on a typical marine radar include gain, 'anti sea-clutter' and 'anti-rain clutter', and 'interference rejection'. These settings are applied to the radar signal returning from the antenna, and are there primarily so that the user can improve the ability to detect targets when they are viewing the radar PPI. Gain adjusts the sensitivity of the screen to the return signal. A high gain will show all return signals (including significant amounts of 'noise') whereas a low gain will remove information on all but the largest targets. Sea anti-clutter changes the slope of the gain function at short ranges, which reduces the strength of the return echo from nearby targets. Anti-rain clutter rain breaks up return echoes from large targets. In addition, some radars have a “sensitivity time control” or STC adjustment, which 'smoothes out' the intense power of the beam at shorter ranges (e.g. Schmaljohann et al. 2008). 

For biological studies that involve post-processing of the radar signal, gain should be high (e.g. probably as high as possible, maximizing the information returned, but ultimately this will be a function of how a particular radar unit deals with gain) and all anti-clutter transformation should be turned off, or the information in the return signal will be altered (usually in some unknown way; Schamljohann 2008). Effectively what is wanted is to maximize the amount of information between the lowest and highest power levels detected by the radar, and for these to be related to the output of the radar digitizing card in a linear (or at least straightforward, and known) manner. 

Finally, an interference rejection setting suppresses interference caused by a nearby radar of the same frequency. Because this function detects and filters out small signal returns (probably in some way unknown to the user) it may also filter out small biological targets and should probably not be used. However, some researchers have used interference rejection when simultaneously running two radars in proximity (e.g. Cooper et al. 1991).

In summary, the user should arrange to digitize the radar signal obtained after the IF carrier has been removed and any subsequent amplification performed, but before any manufacturer-specific "black-box" filtering has been applied. If this is not possible, the user should at least obtain detailed documentation on the effects of any processing that occurs upstream of the point where the radar signal is tapped.  A qualified radar technician can inspect the circuit diagram for the user's radar, and determine the best place to tap the signal which will be fed to the card.. The radR project aims to facilitate sharing of such information among radar users.  As an empirical test of the digitization, the user can change the radar's settings to maximize target sensitivity, and then compare the radar's PPI screen to that generated by the radR software from the raw digitized radar signal.  All features of interest on the radar's PPI should be visible in radR's plot window (possibly after adjusting radR display parameters such as the palette) and typically, radR's plot window will display additional information not available in the radar PPI.

Ground clutter and side-lobe reduction
Ground clutter results from unwanted signals returned from objects on the ground near the radar. Clutter can be generated by either “side lobes” or “spillover radiation” (Larkin 2005).  Side lobes are regions beyond the edges of the main radar beam where transmitted power is significant, but much lower than in the main beam.  Spillover radiation is any energy that escapes the antennas' edge (Larkin 2005).  Both can bounce off nearby structures or vegetation and result in excess energy saturating close ranges (Larkin and Eisenberg 1978) and masking of nearby targets.  Extensive documentation exists on reducing 'clutter' (Eastwood 1967, Williams et al. 1972, Cooper et al. 1991, Bruderer 1997, Larkin 2005); clutter reduction is usually critical to obtaining useful radar data at short ranges, but is usually site and radar specific. 

Clutter can be reduced in a variety of ways. It tends to be less of a problem when the angle of the radar antenna from the ground is higher, since the proportion of the beam that is intersecting local structures is reduced (Eastwood 1967, Cooper et al. 1991). Similarly, raising the radar off the ground on a tower or vehicle top (Cooper et al. 1991, Harmata et al. 2003) can also reduce clutter. Alternatively (or in addition) some of the side-lobe or spillover energy can be blocked by situating the radar in a shallow depression (Eastwood 1967) or amid vegetation (Williams et al. 1972). Researchers have also constructed radar fences by attaching a metal lip to the lower side of an open array (Cooper et al. 1991), attaching a collar to a parabolic dish (Cooper et al. 1991), or constructing a circular barrier around the antenna using radar absorbent material (Larkin 2005).  Aluminum screening may partially absorb radar energy provided the mesh size is no more than half the wavelength (i.e. 1.6 cm for marine X-band radars). It should be noted that all physical anti-clutter devices likely alter the empirical shape of the beam to some extent which has implications for interpretation. 

Where to put the radar
The radar should be placed in a location where the antenna can scan the volume of sky from which the user is interested in obtaining information. Although this may seem trivial, several things are important: a clear view of the volume being scanned and positioning the actual beam so that it is close enough to the volume that meaningful information can be obtained on enough targets (e.g. a consideration of the detection probability and radar range). Since radar energy reflects well from water and metal, users should position radars away from metal buildings and avoid directing the beam at too low an angle when near open water (especially under wavy conditions).

3) Acquiring the signal from the radar. 
Several methods of collecting radar data have been used in the past, including film (Richardson 1978a), acetate sheets overlain on the PPI (Harmata et al. 1999) and video-frame grabbing software. However, to obtain the maximum amount of information from the radar signal, one must use a specialized analog-digital converter coupled with a computer, must be used. Such cards have been manufactured by researchers (e.g. VSUs; Larkin & Eisenberg (1978); Bruderer et al. (1995)) but are also available commercially, and are known as 'Radar digitizing cards'. These  work by digitizing four analog signals from the radar: (1) "video", which is an analog signal whose voltage represents the power echoed back to the radar from targets; (2) trigger, which indicates exactly the point in time when each pulse begins to leave the radar transmitter, and so provides the reference point for timing echos from targets  (3) heading (or azimuth reset pulse (ARP)), which marks each time the radar has completed a 360 degree rotation and is pointing "forward" again, and (4) bearing (or azimuth change pulse (ACP)), which “ticks” at a fixed angular rate as the antenna rotates, and allows for correction of variability in antenna rotation speed from such sources as power supply fluctuations and the force of wind.

Capturing and processing the raw signal allows the user to obtain considerably more information about biological targets than is typically displayed on standard radar PPI. We will not discuss other ways of capturing the radar signal here, but note that a typical display on a commercial radar unit displays only seven levels of return echo intensity, whereas current commercial models of radar digitizing cards can acquire as many as 4096 levels of intensity (12 bits) from the same signal. We are aware of radar digitizing cards available from several manufacturers; here we present details on the Sigma S6  card from Rutter Technologies Inc. (St. John's, Newfoundland, Canada) which we have used extensively. Other cards, such as the XIR3000 manufactured by Russell Technologies Inc. (North Vancouver, British Columbia, Canada), work in much the same way but may have different sampling bit depths and rates. Users will have to consult specific manuals and radar technicians to deal with differences among cards, and they should also be aware that this is one area where technology is likely to change significantly in the future. In particular, commercial off-the-shelf A/D converters now exist that can handle the high sampling rates and bit depths necessary to convert a raw radar signals to a digital data stream, but require additional programming to deal with the pulsed nature of radar data.

Setting up the card
Card set up procedures vary widely, so we do not provide details here. Users should be aware that they will need to ensure that the card is correctly acquiring and digitizing the data from a single pulse of information, and that it is collecting sufficient pulses from a single scan (i.e. that it is keeping up with the radar input, and not 'dropping' pulses or scans). Manufacturers typically employ some kind of installation program for this purpose, but an empirical test is to compare the data acquired from consecutive pulses – if they remain the same, it is likely that the user is requesting more pulses to be digitized per scan than the card is actually capable of providing on the current hardware platform.  In this case, the user should reduce the requested number of pulses per scan, (because the redundant data is useless), or consider using a more powerful computer or digitizing card. Most importantly, for obtaining biological information, users should attempt to minimize (or preferably eliminate) any processing done by the card (except for the delivery of the digitized pulses of information; see discussion above) and maximize the number of pulses delivered per antenna rotation, subject to hardware and software constraints.

Bruderer, B: The study of bird migration by radar - Part 1: The technical basis. Naturewissenschaften 1997, 84:1-8.

Cohen B, Williams TC: Short-range corrections for migrant bird tracks on search radars. J Field Ornith, 1980. 51:248-253.

Desholm, M: How much do small-scale changes in flight direction increase overall migration distance? Journal of Avian Biology 2003, 34:155-158.

Harmata AR, Leighty GR, O'Neil EL. A vehicle-mounted radar for dual-purpose monitoring of birds. Wildlife Society Bulletin 2003, 31:882-886.

Harmata AR, Podruzny KM, Zelenak JR, Morrison ML. Using marine surveillance radar to study bird movements and impact assessment. Wildlife Society Bulletin 1999. 27:44-52.

Huppop O, Dierschke J, Exo K, Fredrick E, Hill R. Bird migration studies and potential collision risk with offshore wind turbines. Ibis 2006, 146:90-109.

Kahlert J, Petersen IK, Fox AD, Desholm M, Clausager I. Investigations of birds during construction and operation of Nysted offshore wind farm at Rodsand.  Annual status report 2003.  NERI report for Energy E2 A/S. Rønde, Denmark, 2004..
 
Krijgsveld KL, Lensink R, Schekkerman H, Wiersma P, Poot MJM, Meesters EHWG, Dirksen S. Baseline studies North Sea wind farms:  fluxes, flight paths and altitudes of flying birds 2003-2004. Netherlands: Bureau Waardenburg bv, Culemborg; 2005.

Larkin RP, Eisenberg L. A method for automatically detecting birds on radar. Bird-Banding, 1978; 49:172-181.

Peckford ML, Taylor PD. Within night correlations between radar and ground counts of migrating songbirds. Journal of Field Ornithology, 2008; 79:207-214

Petersen IK, Christensen TK, Kahlert J, Desholm M, Fox AD. Final results of bird studies at the offshore wind farms at Nysted and Horns Rev, Denmark. NERI report for DONG energy and Vattenfall A/S.  Rønde, Denmark. 2006

Richardson WJ. Autumn landbird migration over the western Atlantic Ocean as evident from radar. Proceedings of the 17th Int. Ornithological Congress: xx-yy 19xx, Berlin; Edited by …. 1978: 501-506.

Ruth JM. Applying radar technology to migratory bird conservation and management: strengthening and expanding a collaborative: U.S. Geological Survey 2007. Report number 2007-1361. 86 p.

Tulp I, Schekkerman H, Larsen JK, van der Winden J, van der Haterd RJ, van Horssen WP, Dirsken S, Spaans AL. Nocturnal flight activity of sea ducks near the windfarm Tuno Knob in the Kattegat.  Prepared for Novem, Ultrecht, Netherlands. Bureau Waardenburg bv, Culemborg, Netherlands. 1999

Williams TC, Settel J, O'Mahoney P, Williams JM. An ornithological radar. American Birds 1972; 26:555-557.
